# Death from Ether and Chloroform

**Published:** 1878-03

**Authors:** Henry Van Buren


### DEATH FROM ETHER AND CHLOROFORM.

By Henry Van Buren, M. D.

*(Reported to the Chicago Medical Society, January 21st.)*

Mrs. B——, aged 32, American, had suffered from fistulæ in ano for six months. On the 22d of November last, I operated on her, finding at this time two artificial openings into the rectum, one on either side of the anus. Dr. A. Groesbeck administered the anæsthetic, which consisted of equal parts of sulphuric ether and chloroform. The operation was performed in a few seconds. The patient exhibited no alarming symptoms while under the influence of the anæsthetic, and revived in the usual time.

On the morning of the 30th of November, eight days after the operation, I desired to make a thorough examination of the wounds and renew the dressing, and in this, as in some of the previous dressings, the patient insisted upon partial immunity from pain. To this end I commenced to administer upon a napkin two parts of sulph. ether and one of chloroform. After a few inhalations the patient became violently intoxicated, and resisted with great force all efforts to quiet her, demanding in the language of one in delirium, to be let alone. I immediately ceased to administer the anæsthetic, and with great effort prevented her from jumping from the bed. The face became at first turgid, the whole body convulsive, and in a few seconds the patient was dead.

All of the means usually resorted to, were employed to restore action of the vital functions ; artificial respiration, elevating the lower extremities, dashing cold water in the face, drawing forward the tongue, spirits of ammonia applied to the nostrils, and finally a galvanic battery, which was conveniently at hand, but to no avail.

I have to say in justice to the record of this case, that the patient had for many years habitually partaken of opium. At the time of her unfortunate death, she could take at each dose, from two to three grains of morphia. During the time she was under my care, one half grain doses of morphia were prescribed at proper intervals, but she asserted that this quantity did not sufficiently support her, and through her nurse and by stealth, she secured additional quantities from the neighboring drug stores, and took the same daily without my knowledge or consent.

I am now of the opinion that the patient had taken an unusally large dose of morphia on the morning of her death ; and that the combined influence of this overdose, and the additional paralyzing effects of the anæsthetic caused cardiac syncope, and that this was the cause of death.

Whether there was structural lesion of the heart or not I am unable to state, as no post mortem examination was made in this case, and I had suspected none in my previous knowledge of the patient.

There are one or two criticisms which may be made by members of this society, and as I anticipate them, I may be permitted to say in advance, what is to be said in support of the points probably in issue.

Among medical men in the United States at the present day, there is a pretty general belief that sulphuric ether alone is the safer anæsthetic. The belief, I think, is more the outgrowth of popular opinion than of any scientific knowledge of fact.

In England, where there are certainly men who are the peers of members of the medical profession in our own country, no such preference exists for ether, but rather for chloroform.

The *London Lancet*, of November 17th, contains a report of a death at Lincoln, England, from pure sulphuric ether, which occurred in the practice of Dr. G. M. Lowe. The anæsthetic was given with great care, under the most favorable circumstances, and that too with only a few inhalations. This case is reported also in the *Med. Record* of New York, Jan 5th, 1878.

A member of this society reported a case of death from ether less than a year ago, which occurred in his hands in the Illinois Eye and Ear Infirmary.

It was the practice of Dr. Daniel Brainard, up to the time of his death, to use as an anæsthetic equal parts of chloroform and ether.

Dr. Moses Gunn personally informed me that, although he now used ether, for twenty years he had used chloroform only, and that, without a single death.

In 9,000 of the earliest cases of its inhalation at St. Bartholomew's Hospital, it is reported that there was not one death.

M. Fleureur states that it was administered without one casuality to 25,000 French soldiers in the Crimean war.

I do not, however, present these facts in support of any claim that chloroform is the best agent. It no doubt has some advantages and other disadvantages, and it is possible that ether is the safer of the two. As my case stands, it is two against ether and one against chloroform, unless the combination acts some important part.

Again, it may be said that an anæsthetic should not have been given to a patient under the influence of extraordinary doses of opium. To this, I reply that I was not aware that more than half grain doses had been taken. Nor am I ever able to determine, to any degree of nicety in confirmed opium eaters, the amount they may have taken.

My conclusions are these: That while an ordinary dose of opium or stimulant is undoubtedly in some cases an advantage, prior to administering an anæsthetic, ether or chloroform is given with more or less hazard in habitual opium eaters.

---

The Influence of Bromide and Iodide of Potassium on Gastric Digestion.—Dr. Putzeys, after a series of experiments in artificial digestion, in which he substituted hydrobromic and hydriodic acids for hydrochloric acid, concludes *Bulletin de l' Acad. de Belgique*, 1877, xi.) that hydriodic acid, whatever its proportion in the digestive fluid, cannot replace hydrochloric acid, because its action is more feeble and slower. Moreover, he believes that the iodide and bromide of potassium will not be received with equal tolerance if they are ingested at the time of gastric activity; hence it is in every respect preferable to administer these salts, and especially the iodide, a half hour or an hour before meals, when the stomach is empty and its surface is covered with a layer of neutral or even alkaline mucus.

Changes in the Medical Staff of Cook County Hospital.—Dr. H. A. Johnson lately resigned his position of attending physician at the Cook County Hospital, and under the new rules, was transferred upon the consulting staff. The Medical Board assigned Dr. Johnson's place to Dr. Wm. Quine, heretofore one of the gynæcologists of the hospital, and nominated Dr. Norman Bridge to succeed Dr. Quine. This nomination is now in the hands of the conference committee, to be submitted to the County Board for approval.

Germany has 4,416 drug stores, or about two to every 100 square miles, and only one to 10,000 inhabitants.

